# Strengthening and implementing health technology assessment and the decision-making process in the Region of the Americas

**DOI:** 10.26633/RPSP.2017.165

**Published:** 2017-12-26

**Authors:** Fernanda Lessa, Francisco Caccavo, Stephanie Curtis, Stéphanie Ouimet-Rathé, Alexandre Lemgruber

**Affiliations:** 1 Medicines and Health Technologies Unit Pan American Health Organization/World Health Organization Washington, D.C. United States of America Medicines and Health Technologies Unit, Pan American Health Organization/World Health Organization, Washington, D.C., United States of America.

**Keywords:** Technology assessment, biomedical, decision making, health systems, health economics, health priorities, Americas, Evaluación de la tecnología biomédica, toma de decisiones, sistemas de salud, economía de la salud, prioridades en salud, Américas

## Abstract

**Objective.:**

Health technology assessment (HTA) has been adopted by countries in order to improve allocative efficiency in their health systems. This study aimed to describe and analyze the HTA decision-making process in the Region of the Americas.

**Methods.:**

A literature review was done to better understand the HTA situation in the Region. Also, in 2014 and 2015, individuals responsible for conducting HTA in countries of the Americas were identified and received a questionnaire on HTA and the decision-making process.

**Results.:**

A total of 46 questionnaire responses were obtained, from 30 countries. The respondents were similar in terms of their institutions, main funding sources, and technology types assessed. Of the 46 respondents, 23 (50%) work for their respective ministry of health. Also, 36 (78%) undertake and/or coordinate HTA through coverage and reimbursement/pricing decisions and other HTA-related activities, while 24 (52%) use HTA for emerging technologies. While some countries in the Region have created formal HTA units, there is a weak link between the HTA process and decision-making. Most of the countries with recognized HTA institutions are members of the Health Technology Assessment Network of the Americas (RedETSA). Despite the advances in the Region overall, most countries in Central America and the Caribbean are still at the early stages of implementing HTA to support decision-making.

**Conclusions.:**

Many countries in the Americas have benefited from the exchange and capacity-building opportunities within RedETSA. However, there are still many challenges to overcome in the Region in terms of the discussion and creation of HTA-related policies.

## RESUMEN

**Objetivo.:**

Muchos países han adoptado la evaluación de tecnologías sanitarias (ETS) para mejorar la eficiencia distributiva en sus sistemas de salud. Este estudio tuvo por objeto describir y analizar el proceso de toma de decisiones basado en ETS en la Región de las Américas.

**Métodos.:**

Se hizo una revisión bibliográfica para comprender mejor la situación de la ETS en la Región. Además, en el 2014 y el 2015 se identificaron las personas responsables de realizar la ETS en los países de la Región, quienes recibieron un cuestionario sobre las ETS y el proceso de toma de decisiones.

**Resultados.:**

Se recibieron 46 cuestionarios respondidos en total, procedentes de 30 países. Los encuestados eran similares en cuanto a sus instituciones, principales fuentes de financiamiento y tipos de tecnología que evaluaban. De los 46 encuestados, 23 (50%) trabajan para el ministerio de salud de su país. Además, 36 (78%) realizan o coordinan la ETS mediante decisiones relativas a la cobertura y los reembolsos o fijación de precios, mientras que 24 (52%) usan la ETS para las tecnologías emergentes. Si bien algunos países de la Región han creado unidades formales de ETS, la vinculación entre el proceso de la ETS y la toma de decisiones es débil. La mayoría de los países que cuentan con instituciones reconocidas de ETS son miembros de la Red de Evaluación de Tecnologías Sanitarias de las Américas (RedETSA). No obstante el progreso de la Región en términos generales, la mayoría de los países de Centroamérica y el Caribe se encuentran todavía en las fases iniciales de la aplicación de la ETS para apoyar la toma de decisiones.

**Conclusiones.:**

Muchos países de la Región de las Américas se han beneficiado de las oportunidades de intercambio y formación de capacidad que brinda RedETSA. Sin embargo, persisten muchos retos que es necesario superar en la Región en torno al debate y la formulación de políticas relacionadas con la ETS.

## RESUMO

**Objetivo.:**

A avaliação de tecnologias em saúde (ATS) foi adotada pelos países visando melhorar a eficiência alocativa dos próprios sistemas de saúde. O propósito deste estudo foi descrever e analisar o processo decisório de ATS na Região das Américas.

**Métodos.:**

Realizou-se uma revisão da literatura científica para aprofundar o conhecimento sobre a situação da ATS na Região. Em 2014 e 2015, os responsáveis pela ATS nos países das Américas foram identificados e um questionário sobre ATS e o processo decisório foi aplicado”.

**Resultados.:**

Ao todo, foram respondidos 46 questionários provenientes de 30 países. Os participantes apresentavam semelhanças quanto às instituições de trabalho, principais fontes de financiamento e tipos de tecnologias avaliadas. Dos 46 participantes, 23 (50%) trabalhavam no ministério da Saúde do país; 36 (78%) executavam e/ou coordenavam a ATS tomando decisões sobre cobertura e reembolso/precificação e realizando outras atividades relacionadas e 24 (52%) realizavam a ATS de tecnologias emergentes. Embora tenham sido instituídas unidades formais de ATS em alguns países na Região, o vínculo entre o processo de ATS e a tomada de decisão é frágil. A maioria dos países que reconhecidamente instituíram a ATS integra a Rede de Avaliação de Tecnologias em Saúde das Américas (RedETSA). Apesar do progresso na Região, a maior parte dos países na América Central e Caribe ainda está nos estágios iniciais de implementação da ATS para apoiar o processo decisório.

**Conclusões.:**

Muitos países nas Américas se favorecem com o intercâmbio e as oportunidades de capacitação da RedETSA. Porém, a Região ainda precisa superar muitos desafios em termos do debate e formulação de políticas em torno da ATS.

Health technology is a term widely used to refer to different areas of health, including prevention, diagnosis, and therapy. It includes all products used in health services delivery, procedures, and systems (1-3). Health technology assessment (HTA) is a multidisciplinary process for the systematic evaluation of properties, effects,and impacts of health care technology. HTA also considers the clinical, social, ethical, and economic aspects in order to better inform health policymakers and improve the decision-making process in the area (3, 4).

The use of HTA has increased worldwide, enabling coverage decisions to be evidence-based and improving efficiency in resource allocation. The assessment takes into account several aspects, such as efficacy, safety, efficiency, social and legal issues, and ethics (3-7). Technology assessment can be carried out at any point during the product’s life cycle, and it can serve different purposes. These include advising a regulatory agency about the authorization and use of a technology, supporting coverage decisions, advising clinicians and patients about the proper use of a health technology, and guiding disinvestment decisions (8).

Recent years have seen considerable growth in the availability of drugs, diagnostic tools, telemedicine, and surgical equipment. These changes are often associated with positive results, such as improvements in health, quality of life, treatment, organization, and delivery (4, 5). However, the arrival of new technologies and drugs has also produced negative repercussions. The increasing cost of health technologies, exacerbated by public expenditure constraints, is a reality that threatens health care systems in many countries (9). In a number of cases, health expenditures have grown faster than the gross domestic product (GDP) has, leading to difficult compromises between rising patient expectations and limited resources. As a result, decisionmakers are constantly seeking to enhance efficiency. According to an assessment performed by the medical journal *Prescrire* in 2015, only 8 out of the 87 new drugs assessed were described as “a real advance” or “offers an advantage” (9). The remaining 79 drugs tested were either damaging to health or could not be proven safe, due to insufficient documentation (9). Despite the appeal of new technologies and products, assessment is crucial in order to evaluate the value that a technology adds.

## Health technology assessment in the Region of the Americas

In the Americas, the health sector reforms of the 1990s encouraged health equity and inclusion by establishing legal rights to health protection. Indeed, many countries have established social and welfare reforms to reduce poverty and expand access to nutrition, education, and health. The Region’s health systems have used social health insurance or tax-based financing to extend health care services through benefit packages (10, 11). This push toward achieving universal health reinforces the role of HTA in Latin America and the Caribbean, especially in a period when health systems are under pressure from increasing costs and declining budgets (11).

Recognizing the importance of this issue, in September of 2012, the Member States of the Pan American Health Organization (PAHO) adopted Resolution CSP28.R9, titled Health Technology Assessment and Incorporation into Health Systems. The resolution encouraged Member States to establish decision-making processes to incorporate health technologies based on HTA; to use HTA to inform public health policies, including public health system coverage decisions; to develop clinical guidelines and protocols for new technologies; and to actively participate in the Health Technology Assessment Network of the Americas (RedETSA) (3, 6). RedETSA has 33 members in 16 countries: Argentina, Bolivia, Brazil, Canada, Chile, Colombia, Costa Rica, Cuba, Ecuador, El Salvador, Honduras, Mexico, Panama, Paraguay, Peru, and Uruguay. PAHO serves as the secretariat of the organization (6). Passage of that resolution was one in a series of recent advances in the institutionalization of HTA in the Americas, which will be discussed later in this article.

The aim of this article is to describe and analyze the HTA decision-making process in the Region of the Americas and to establish a baseline for future impact assessments in the Region.

## METHODS

This study was part of a project named Advance-HTA and was funded by the European Commission’s 7^th^Framework Programme (FP7/2007-2013) under grant agreement No. 305983 (http://www.advance-hta.eu/). The study was done in two parts, a literature review and a survey.

The structured literature review was done to find all relevant articles related to HTA in the Americas, in order to develop understanding and insight into the current situation in the Region. The literature search included the MEDLINE (PubMed) and LILACS (BIREME) databases (Annex 1).

The selected countries included in the literature review were Antigua and Barbuda, Argentina, Aruba (as a PAHO Associate Member), the Bahamas, Barbados, Belize, Bermuda, Bolivia, Brazil, Canada, Chile, Colombia, Costa Rica, Cuba, Curaçao (as a PAHO Associate Member), Dominica, the Dominican Republic, Ecuador, El Salvador, Grenada, Guatemala, Guyana, Haiti, Honduras, Jamaica, Mexico, Nicaragua, Panama, Paraguay, Peru, Saint Kitts and Nevis, Saint Lucia, Sint Maarten (as a PAHO Associate Member), Saint Vincent and the Grenadines, Suriname, Trinidad and Tobago, Uruguay, and Venezuela.

We excluded papers published before 2000 and also ones written in a language other than English, Portuguese, or Spanish. All the articles selected included information about decision-making processes, HTA capacity, and the different uses of HTA to inform decisions in the countries of the Americas.

From March to July of 2014, a cross-sectional study was performed in the countries and territories selected for the Advance-HTA project in the Region of the Americas: Bahamas, Barbados, Belize, Bermuda, Costa Rica, Dominica, Dominican Republic, El Salvador, Guatemala, Guyana, Haiti, Honduras, Jamaica, Nicaragua, Panama, Saint Lucia, Sint Maarten, Suriname, Trinidad and Tobago, and Venezuela. PAHO focal points in each country were asked to identify the individuals and/or institutions responsible for conducting HTA in their countries and to send them invitations to participate in the study. If no individual or institutions were identified, the PAHO focal point in that country was then invited to participate in the study and receive the survey. RedETSA focal points in Costa Rica and El Salvador also received the questionnaire.

Potential participants received both a PDF copy of the questionnaire (titled “HTA and the Decision-Making Process in Emerging Settings”) and a Survey Monkey Web hyperlink to the same questionnaire. The questionnaire was divided into five sections: i) Introduction/Country Settings; ii) Use of HTA in the Country; iii) Decision-making Process; iv) Implementation of the Decision; and v) HTA and Decision-making: Future Challenges. A copy of the questionnaire is available from the authors.

The survey had been piloted in three Caribbean countries from January to February 2014. And, before that, the survey had been sent to the London School of Economics and to NICE International for feedback on its content and wording. (Established in 2008 by the United Kingdom’s National Institute for Health and Clinical Excellence (NICE), NICE International offers advice to governments and governmental agencies overseas on building capacity for assessing and interpreting evidence to inform health policy and on designing and using methods and processes to apply this capacity to their local country setting.)

In order to obtain a more complete overview of the Region of the Americas, during February through September of 2015, the survey was sent to these other PAHO Member States: Argentina, Bolivia, Brazil, Canada, Chile, Colombia, Costa Rica, Cuba, Ecuador, Mexico, Paraguay, Peru, and Uruguay.

All contact with survey respondents was made via email. As needed, email reminders were sent to possible respondents, asking them to complete the survey. In addition, when there were contradictory answers provided by different respondents from the same country, the respondents were contacted for clarification. The identity of all the respondents has been kept confidential.

## RESULTS

### Literature search

For the literature search, PubMed and LILACS were used in order to obtain a better idea of the range of publications on HTA and decision-making in the Region of the Americas. Annex 1 shows the number of references found for each country, before the exclusion process. Among the countries in the Americas, Canada, Brazil, Argentina, Mexico, Colombia, and Chile have published the most on the subject.

After excluding duplicates and search items that were not related to the study’s objectives, as indicated by their title or abstract, a total of 226 articles were selected. After the full-text reading, a total of 137 references were considered.

### Survey on the decision-making process

Responses from 30 of 38 countries and territories were obtained, with a total of 47 questionnaires received. One respondent had left the survey blank, so 46 answers were considered valid (Table 1). Antigua and Barbuda, Aruba, the Bahamas, Curaçao, Dominica, Grenada, Saint Kitts and Nevis, and Saint Vincent and the Grenadines did not participate in this study. The country with the most responses was Costa Rica (5), followed by Argentina and Brazil, with 4 responses each, and Colombia, with 3 responses.

Out of the 46 respondents, 26 of them (57%) were institutional member of RedETSA. As shown in Table 2, there is homogeneity in the characteristics of the respondents. Most belong to similar institutions, share comparable sources of funding for HTA, and assess the same type of technology. Twenty-three (50%) work for their respective ministry of health (MoH), 5 (11%) for a regulatory agency, and 4 (9%) for social security. Fourteen (30%) stated that they work for some other institution, such as a hospital, university, or international health organization.

The most common activity performed by respondents was undertaking HTA (37%), followed by the coordination of HTA activities (28%) (Table 2). Other activities included coverage/reimbursement decisions (13%), pricing decisions (13%), the development of clinical guidelines based on HTA (13%), and other things (17%). The use of HTA throughout a technology’s life cycle was concentrated in emerging technologies (52%), followed by established or widespread practice (30%). HTA was less commonly used for technology with declining use (9%).

In the region of the Americas, 13 of the 30 countries that responded to the questionnaire have established HTA bodies or organizations: Argentina, Bolivia, Brazil, Canada, Chile, Colombia, Costa Rica, Ecuador, El Salvador, Mexico, Peru, Uruguay, and Venezuela. (Bolivia established its national HTA committee after the initial questionnaire period, while this article was being prepared.) For Canada, the only respondent was a representative from INESSS, an independent organization that reports to Quebec’s Minister of Health and Social Services. All the respondents with established HTA bodies or organizations are RedETSA members, except for Venezuela.

Of the 46 respondents, 39 of them (85%) reported carrying out assessments on pharmaceuticals, followed by medical devices (n = 33; 72%), and medical procedures (n = 22; 48%). Of the 46 respondents, 23 (50%) answered that there are no guidelines on how to perform HTA in their country, and 31 (67%) used HTA reports that had been carried out in other countries to guide their decision-making. Furthermore, 59% (n = 27) answered that there is no legislation in place to ensure that decision-making is supported by HTA. Despite this, 16 out of those 27 respondents (59%) without official legislation in place reported that HTA findings are still used in decision-making processes. Of the 46, 18 (39%) also stated that there are no mechanisms for monitoring or evaluating the HTA recommendations.

Cost-effectiveness thresholds were identified in 5 of the 30 countries. In Costa Rica, Ecuador, and Peru, the cost-effectiveness threshold is estimated to be three times the gross domestic product (GDP) per capita, whereas in Chile and Mexico, it is one times the GDP per capita. Chile’s value was officially established in the Chilean Economic Evaluation Methodological Guideline, published in 2013.

When questioned about the main barriers that arise in the HTA decision-making process in their countries, 18 of the 46 respondents (39%) mentioned organizational/institutional barriers, 14 (30%) a lack of human resources, and 12 (26%) a lack of funding.

**FIGURE 1.**
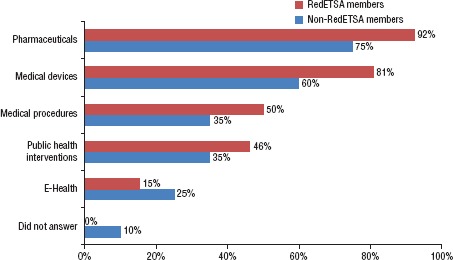
Health technologies assessed in the Region of the Americas among countries that are members of the Health Technology Assessment Network of the Americas (RedETSA) (n = 26) and those that are not members (n = 20), 2014-2015

***Source:*** Prepared by the authors, based on data from the study, 2014-2015.

As shown in Figure 1, for both RedETSA members (n = 26) and nonmembers (n = 20), pharmaceuticals are the most assessed technologies (92%, n = 24, and 75%, n = 15, respectively), followed by medical devices (81%, n = 21; 60%, n = 12).

Among the 26 respondents that are members of RedETSA, 18 of them (64%) stated that there are methodological guidelines for conducting HTA in their countries. In addition, 14 of the 26 (54%) reported that there is no legislation to ensure that decision-making is supported by HTA. However, of those 14 that reported there is no such legislation, 11 of them (79%) reported using HTA findings despite there being no formal legislation, and 10 of the 26 (38%) stated they had some mechanisms for monitoring and evaluating the HTA recommendations. Of the 26, 21 of them (81%) stated that they use HTA reports developed in other settings in their decision-making processes.

**TABLE 1.** Respondents in the Americas (N = 46) to survey on health technology assessment and the decision-making process, 2014-2015, with country, number of respondents, institution, and status of membership in the Health Technology Assessment Network of the Americas (RedETSA)

Country	No. of respondents	Institution that participated in the study	RedETSA member?
Argentina	1	Dirección de Economía de la Salud, Ministerio de Salud de la Nación (MSAL)	Yes
Argentina	1	Hospital Garrahan/RedArets	Yes
Argentina	1	Instituto de Efectividad Clínica y Sanitaria (IECS)	Yes
Argentina	1	Dirección de Calidad en los Servicios de Salud, Ministerio de Salud de la Nación (MSAL)	Yes
Barbados	1	Barbados Drug Service (BDS) of the Ministry of Health	No
Belize	1	PAHO Country Office	No
Bermuda	1	Bermuda Health Council and Ministry of Health and Environment	No
Bolivia	1	Ministerio de Salud, Unidad de Medicamentos y Tecnología en Salud	Yes
Brazil	1	Agência Nacional de Vigilância Sanitária (Anvisa)	Yes
Brazil	1	Instituto de Engenharia Biomédica/Universidade Federal de Santa Catarina	Yes
Brazil	1	Instituto Nacional de Cardiologia (INC)	Yes
Brazil	1	Departamento de Gestão e Incorporação de Tecnologias em Saúde, Ministério da Saúde (DGITS/MS)	Yes
Canada (Quebec)	1	Institut national d’excellence en santé et en services sociaux (INESSS)	Yes
Chile	1	Instituto de Salud Pública de Chile (ISP)	Yes
Chile	1	Ministerio de Salud	Yes
Colombia	1	Instituto de Evaluación Tecnológica en Salud (IETS)	Yes
Colombia	2	Ministerio de Salud y Protección Social	Yes
Costa Rica	2	Caja Costarricense de Seguro Social (CCSS)	Yes
Costa Rica	1	Hospital La Católica	No
Costa Rica	1	Ministerio de Salud	Yes
Costa Rica	1	Universidad de Costa Rica	No
Cuba	1	Ministerio de Salud Pública	Yes
Dominican Republic	1	Ministerio de Salud Publica	No
Dominican Republic	1	PAHO Country Office	No
Ecuador	1	Ministerio de Salud Pública	Yes
El Salvador	1	Ministerio de Salud	Yes
Guatemala	1	PAHO Country Office	No
Guyana	1	Ministry of Health	No
Haiti	1	Ministère de la Santé Publique et de la Population (MSPP)	No
Honduras	1	PAHO Country Office	No
Jamaica	1	Ministry of Health	No
Mexico	1	Centro Nacional de Excelencia Tecnológica en Salud (CENETEC)	Yes
Nicaragua	1	PAHO Country Office	No
Panama	2	Caja de Seguro Social (CSS)	No
Paraguay	1	Instituto de Investigaciones en Ciencias de la Salud/Ministerio de Salud Pública y Bienestar Social	Yes
Peru	1	Instituto Nacional de Salud del Perú	Yes
Saint Lucia	1	Ministry of Health	No
Sint Maarten	1	Ministry of Public Health, Social Development and Labor	No
Suriname	1	Ministry of Health	No
Trinidad and Tobago	1	PAHO Country Office	No
Uruguay	1	Ministerio de Salud Pública (MSP)	Yes
Uruguay	1	Fondo Nacional de Recursos (FNR)	Yes
Venezuela	1	Ministerio del Poder Popular de la Salud	No

***Source:*** Prepared by the authors, based on data from the study, 2014-2015.

In contrast, with the 20 respondents from non-RedETSA institutions, 13 of them (65%) reported the absence of methodological guidelines for conducting HTA. While 16 of the 20 (80%) reported there is no legislation to ensure that decision-making is supported by HTA, 4 of those 16 (25%) stated that they used HTA findings to support decision-making. Of the 20, 8 of them (40%) reported not having any mechanisms for monitoring and evaluating the HTA recommendations, 4 of them (20%) stated they have some mechanism, and the other 8 respondents (40%) were unsure or did not respond. Of the 20, 8 of them (40%) indicated that they use HTA reports developed in other settings in their decision-making processes. The nonresponse rate for this question was significantly higher for non-RedETSA members (40%) than for RedETSA members (0%), a point that will be addressed later in this article.

**TABLE 2.** Characteristics of the respondents in the Americas (N = 46) to the survey on health technology assessment (HTA) and the decision-making process, 2014-2015

Characteristic	No.	%
Institution to which belong		
Ministry of health	23	50
Regulatory agency	5	11
Social security	4	9
Other^a^	14	30
Activities performed		
Undertakes HTA	17	37
Coordinates HTA activities	13	28
Coverage/reimbursement decisions	6	13
Pricing decisions	6	13
Develop clinical guidelines based on HTA	6	13
Other	8	17
Point in a technology’s life cycle at which HTA is used		
Emerging technologies	24	52
Established or widespread practice	14	30
Technology with declining use in practice	4	9
Not Answered	4	9

***Source:*** Prepared by the authors, based on data from the study, 2014-2015.

a Categories of other institutions were: PAHO focal points, 4; hospitals, 3; academia, 2; other institutions, 5.

**FIGURE 2.**
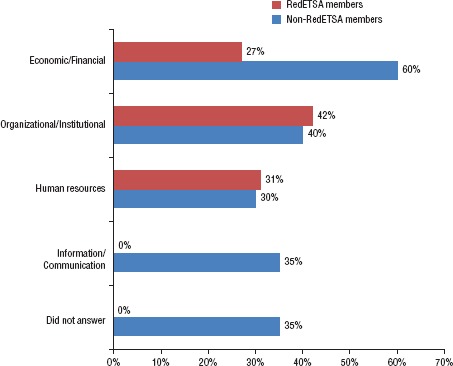
The main barriers in the decision-making process on health technology assessment (HTA) in the Region of the Americas among countries that are members of the Health Technology Assessment Network of the Americas (RedETSA) (n = 26) and those that are not members (n = 20), 2014-2015

***Source:*** Prepared by the authors, based on data from the study, 2014-2015.

The main barriers that arise in the decision-making process for HTA were also analyzed from the perspective of RedETSA membership (Figure 2). Among the 26 respondents that were members of RedETSA, 11 of them (42%) stated that organizational/institutional barriers are the main impediments to decision-making, followed by a lack of human resources (8 of 26, or 31%) and a lack of financial resources (7 of 26, or 27%). For the 20 non-RedETSA members, the most common barrier reported was lack of financial resources (12 of 20, or 60%), followed by organizational/institutional barriers (8 of 20, or 40%) and human resources (6 of 20, or 30%). All the RedETSA members responded to this question, while the non-RedETSA members had a noticeably high nonresponse rate of 35%.

## Discussion

Our literature review showed that scientific production on HTA and the decision-making process has been scarce for Central American and Caribbean countries, but widespread in Canada and Brazil.

Although advances have occurred in the Region of the Americas in the discussion and formulation of policies related to the HTA process, there are still many obstacles to overcome, as indicated in Figure 2. Among the respondents that were not RedETSA members, a high percentage of them did not answer the survey question on the main barriers in the decision-making process on HTA. This could be explained by the fact that they are in the early stages of HTA implementation and/or that the HTA-related questions were not applicable to their current context.

Despite the substantial progress seen in recent years in the Region, in some countries the implementation of HTA remains at a low level. Of the 30 countries that participated in the study, 25 said they performed some kind of HTA-related activity, but only 13 of them have officially established formal HTA bodies, such as an agency, institute, commission, or unit (Table 3). Institutionalization is essential for highlighting obligations and ethical responsibilities, establishing transparent processes, and identifying stakeholders involved in the chain that empowers decisionmakers. All of the formal HTA bodies, except the one in Venezuela, are members of RedETSA. RedETSA seeks to strengthen, increase, and promote the evaluation process for technologies in the Americas and to allow the exchange of information. These kinds of assistance from RedETSA could explain the significant improvements made by some of the countries in the Region.

The involvement of stakeholders and governance and decision-making bodies can substantially affect the impact of HTA. As indicated in Table 3, countries without an official HTA entity can still have HTA activities performed through universities, institutes, nongovernmental organizations, hospitals, and independent units that appreciate the value that these analyses add to the health system. For example, in Argentina, the IECS, an independent HTA institute, conducts HTA for private and public insurance systems throughout the country. In Brazil, Anvisa uses HTA for pricing decisions in the health care system, while the INC applies HTA for decision-making at the hospital level (Table 1). HTA can also be used to advise or inform clinicians and patients about the correct use of health technology (8), as INESSS does in Canada through a number of guideline documents (http://www.inesss.qc.ca/en/publications/inessss-guides.html). In Panama, although there is no official HTA body, the CSS carries out some HTA activity for decisions on pricing, coverage, and reimbursement in its own system. In Paraguay, the health ministry conducts systematic reviews for the development of applications for decision-making in telemedicine.

Our study showed that there is a gap between the conclusions reached through HTA and decision-making in the Region of the Americas. Despite most countries performing some kind of HTA activity, 57% of respondents stated that there is no legislation to promote the use of HTA in the decision-making process. The four countries with related legislation are all members of RedETSA: Brazil, Chile, Colombia, and Uruguay. However, Brazil is the only country where the use of HTA in decision-making is mandatory. Thirty-nine percent of respondents indicated that there were no guidelines for conducting HTA in their countries, but 11% did not answer this question. Sixty-two percent said they used HTA reports that had been produced in other jurisdictions to help in their decisions, which agrees with findings from other authors (15-21). HTA work produced in different contexts is usually adapted to the local situation in order to be used in the decision-making process. When properly done, this saves time and money, prevents inefficiencies and duplication, and enables the transfer of knowledge between different situations (15, 22-25).

The monitoring and evaluation (M&E) of health technology incorporation is also a challenge. Monitoring is defined as the routine tracking and reporting of priority information about a policy, program, or project. It provides information on the level of implementation of each product. Evaluation is the systematic collection of information about these courses of actions (policy/program/project).

In many health care systems, M&E is strategically used to achieve the desired results of a policy, program, or a project by making the correct decisions (26). In our research, 39% stated that there are no M&E mechanisms in place to assess the impact of HTA.

Cost-effectiveness evaluations are used to assess health technologies and help decisionmakers to evaluate what they receive in return for the money they spend on health care. Explicit or implicit thresholds (above which technology is considered cost-ineffective for the health care system) are also sometimes used in decision-making. At the time of our study, Chile was the only country in the Region to have an established threshold in a legal document, of one GDP per capita (27). However, by the time this paper was prepared, Uruguay had approved legislation requiring the use of thresholds for the economic evaluation of medicines. In addition, several countries have adopted an unofficial reference value, including one GDP per capita in Mexico and three GDPs per capita in Costa Rica, Ecuador, and Peru. (In Costa Rica, the threshold only applies to the Central Pharmacotherapy Committee of the Costa Rican Social Security Fund.)

As mentioned earlier in this article, there are barriers to overcome in the Americas for HTA and decision-making. With the survey question regarding barriers, all the RedETSA members answered it, but 35% of the non-RedETSA members did not answer it. For the nonmembers, this might be explained by the lack of awareness concerning the decision-making processes in their countries or their unfamiliarity with HTA issues.

The barriers for implementing HTA are well known. Organizational and institutional difficulties were mentioned by 41% of the respondents, showing that substantial changes related to organizational issues are required. In addition, 30% indicated a lack of human resources, and 4% pointed to insufficient financial means. The HTA process depends on qualified human resources, given the correlation that exists between highly skilled personnel and evidence-based decisions (28). The communications obstacle appears to have been overcome for RedETSA members (with 0% saying it is a barrier), indicating that network’s accomplishments in information sharing.

**TABLE 3.** Overview of established public health technology assessment (HTA) bodies in the Region of the Americas^a^

Country	Established public HTA body	Identified HTA activities in the country
Argentina	HTA unit, Ministry of Health (MoH)	Yes
Barbados	No	Yes^b^
Belize	No	Yes
Bermuda	No	No
Bolivia	Comité Nacional de Evaluación y Uso Racional de Tecnologías en Salud (CNET)	Yes
Brazil	Comissão Nacional de Incorporação de Tecnologias (CONITEC)	Yes
Canada	Canadian Agency for Drugs and Technologies in Health (CADTH); Institut national d’excellence en santé et en services sociaux (INESSS) (for Quebec only)	Yes
Chile	Evaluación de Tecnologías Sanitarias (ETESA)	Yes
Colombia	Instituto de Evaluación Tecnológica en Salud (IETS)	Yes
Costa Rica	HTA commission, Caja Costarricense de Seguro Social	Yes
Cuba	No	Yes
Dominican Republic	No	No
Ecuador	HTA unit, MoH	Yes
El Salvador	HTA unit, MoH	Yes
Guatemala	No	No
Guyana	No	No
Haiti	No	Yes
Honduras	No	No
Jamaica	No	Yes^b, d, e^
Mexico	Centro Nacional de Excelencia Tecnológica en Salud (CENETEC)	Yes
Nicaragua	No	Yes
Panama	No	Yes^b^
Paraguay	No	Yes^f^
Peru	Instituto de Evaluación de Tecnología en Salud e Investigación (IETSI); Instituto Nacional de Salud (INS)	Yes
Saint Lucia	No	Yes
Sint Maarten	No	Yes^b, d^
Suriname	No	Yes^d, e^
Trinidad & Tobago	No	Yes
Uruguay	Ministerio de Salud Pública; Fondo Nacional de Recursos (FNR)	Yes
Venezuela	Comisión Nacional de Evaluación de Tecnología en Salud (CONETS)	Yes

***Source:*** Prepared by the authors, based on data from the study, 2014-2015.

a HTA bodies refer to official HTA institutions that are dedicated specifically to HTA activities or have HTA among their principal activities. However, countries without an official HTA entity can still have HTA activities performed.

b HTA activity: pricing decisions.

c In addition to INESSS (in Quebec), Canada has other HTA provincial bodies not mentioned in this article.

d HTA activity: coverage/reimbursement decisions.

e HTA activity: develop clinical guidelines based on HTA.

f HTA activity: systematic reviews for the development of applications in telemedicine.

### Conclusions

In recent years, there have been clear advances in the institutionalization of HTA in the Region of the Americas, both at a regional and national level (Annex 2). Between the submission of the questionnaires and the preparation of this article, the Region experienced further advances. These included Uruguay’s approval of a cost-effectiveness threshold for the economic evaluation of health technologies, the creation of an HTA institute in Peru, and the establishment of an HTA commission in Bolivia. Furthermore, a proposal for the creation of an HTA agency was submitted to the Senate in Argentina in July 2016 and is expected to be approved. Also, in Mexico, a standard has been approved that defines the linkages between CENETEC, the Price Commission, and the General Health Council, with the aim of improving decision-making.

Despite progress in the discussion and creation of policies concerning HTA processes and HTA institutionalization in the Americas, there is still a lot to be accomplished. The main gap to be addressed is the lack of explicit links between HTA activities and the incorporation of health technologies into health systems.

The main shortcoming in this research was the limited availability of literature on the use of HTA in the decision-making process in the Americas. From the literature review, it was evident that the majority of publications were about countries with a developed HTA structure, such as Argentina, Brazil, Canada, Chile, Colombia, and Mexico (2, 4, 7, 8, 10, 13, 29-46). This shortage of information reinforces the need for research in countries with less developed HTA and decision-making processes.

There are significant differences in the level of implementation of HTA for decision-making in the Americas among the RedETSA members when compared to the nonmembers. Significant progress has been achieved in HTA implementation: some countries have formed HTA units and utilize HTA at some point in the decision-making process. More advances have been made in RedETSA countries than in nonmember countries. This could indicate that RedETSA countries have benefited from the exchange and capacity-building opportunities available through this regional network.

The regional inequality in HTA development highlights the importance of the information gathered on the use of HTA in the decision-making process throughout the Americas. Implementing HTA processes is expected to produce substantial benefits in all countries, especially in Central America and the Caribbean, where most countries are at the early stages of implementing HTA to support decision-making.

### Acknowledgments.

The authors wish to acknowledge and thank all the people who participated in and contributed to this study. This is especially true for the RedETSA members that helped gather and validate the data. We also recognize the substantial improvements in the article’s clarity resulting from the suggestions from the peer reviewers and the work by the *RPSP/PAJPH* technical editor. Funding from the European Commission’s 7^th^Framework Programme is gratefully acknowledged.

### Funding

This study was part of the Advance-HTA project and was funded by the European Commission’s 7^th^Framework Programme (FP7/2007-2013) under grant agreement No. 305983.

### Disclaimer

Authors hold sole responsibility for the views expressed in the manuscript, which may not necessarily reflect the opinion or policy of the *RPSP/PAJPH* or PAHO.
